# Reducing the Risk for Waterborne Nosocomial Neonatal Legionellosis

**DOI:** 10.3201/eid2106.141779

**Published:** 2015-06

**Authors:** Joseph S. Cervia

**Affiliations:** Hofstra–North Shore/Long Island Jewish Health System School of Medicine, Hempstead, New York, USA

**Keywords:** bacteria, legionellosis, neonates, waterborne, nosocomial, *Legionella pneumophila*

**To the Editor:** I read with interest the report by Wei et al. (*1*) regarding 2 cases of neonatal legionellosis associated with infant formula prepared with hospital tap water. Two hospitals were involved, and water samples from both were positive for *Legionella pneumophila* bacteria that had molecular profiles indistinguishable from those for bacteria from the infected neonates. As Wei et al. (*1*) and others have established, control of waterborne pathogens, such as *Legionella* spp., in health care institutions remains a work in progress.

Recently, leading medical centers have recognized the efficacy and cost-effectiveness of performing certain measures to ensure the safety of hospital water. These measures include routine microbial analyses of tap water and use of waterborne pathogen prevention and control measures such as hot water flushing of plumbing; use of chlorination, chlorine dioxide, monochloramine, copper–silver ionization, or ultraviolet light; ozonation; and point-of-use water filtration. Each method has advantages and disadvantages related to ease of implementation, cost, maintenance issues, and short- and long-term effectiveness. Randomized controlled trials comparing the efficacy of these strategies are lacking, but the availability of guidance for using waterborne pathogen prevention and control strategies has resulted in substantial declines in health care–associated legionellosis (*2*). Efforts at waterborne pathogen detection and control are complicated by the role of biofilm, comprising microbes embedded in the polymeric matrix attached to internal plumbing surfaces, which protects waterborne pathogens from adverse environmental conditions, including antimicrobial agents and systemic controls (e.g., ultraviolet light, metals, acid pH) (*2*,*3*).

Prevention of legionnellosis in health care settings offers a clinically beneficial and cost-effective alternative to intermittent case detection and outbreak control. For example, it has been demonstrated that, even in the absence of a recognized outbreak, hospital units caring for immunosuppressed patients can reduce infection rates by using water filtration at the point of use (*4*). Although further efforts are needed to systematically evaluate *Legionella* spp. control measures, a progressive approach to prevent health care–associated legionellosis includes routine microbial analysis of tap water in units for patients at high risk for infection, use of systemic water disinfection technology, and use of point-of-use water filtration in units where care is rendered for patients most vulnerable to infection with *Legionella* spp.
